# 

**DOI:** 10.1017/S0016672320000014

**Published:** 2020-02-17

**Authors:** Beverley Speight

**Affiliations:** Addenbrooke's Hospital, Cambridge University Hospitals NHS Foundation Trust Email: beverley.speight@addenbrookes.nhs.uk

“Families are messy in their structure and their interaction.” This short statement, nested halfway through Chapter 4, is at the core of the newly published *Advanced Genetic Counseling: Theory and Practice* from Oxford University Press. Understanding the social as well as biological factors that underlie how each individual responds to personal genetic information is key work of genetic counselling. This book sets out to demystify the work of Genetic Counsellors and, in doing so, has provided an up-to-date and practical guide for those new and experienced in the field.

The book starts with a history of the intertwined origins of genetic counselling as an activity and Genetic Counsellors as a profession. Later on, counselling and ethical theories that can be successfully used to guide genetic counselling are described. Each counselling theory, its strengths and relevance to genetic counselling are separated out with illustrative effect. Widely familiar approaches, such as Cognitive Behavioural Therapy, and contemporary methods, such as strength-based counselling, are given equal footing, with Genetic Counsellors encouraged to find out in the application what best serves their style and their patients’ needs. Ethical theories are also laid out in accessible ways and applied to well-chosen case scenarios. For clinical practice, there is a consistent message weaved through the theory and evidence: respond to individuals, be wary of assumptions and patterns of response based on the practitioner's experience and preconceptions. Supervision is strongly encouraged.

The case for research evidence-based practice is convincingly made, with helpful ideas for ways in which Genetic Counsellors can be involved or lead this. As the reference lists show, the authors speak with experience and authority on quality research in genetic counselling. This endeavour demands time and collaborative efforts. Often, there can be a tension for clinical Genetic Counsellors between delivering a full-time caseload (and being well placed to pose the pertinent research questions) and a desire to generate patient-centred research data to inform practice. This division of time and commitment is not addressed in the otherwise compelling Chapter 7, ‘Conflict of Interest and the Code of Ethics’.

The book concludes with thoughts from the authors on future directions in Chapter 11, ‘Genetic Counseling in the Genomic Era’. We have moved from a time of few tests and large informational patient needs to vastly expanded tests and a patient population informed by the Internet. Increasingly, pre-test consent is obtained by other health professionals, as Genetic Counsellors move towards a post-test working environment, in which patients are referred with pertinent results. Considerations on the transition, bringing gains and losses and new technology, are given insight in this final chapter.

The information content of genetic counselling changes with the times. The authors are clear on what does not – the ineffectiveness of an educational model, compared to a counselling focus. The latter, advanced by Seymour Kessler's writing in the 1990s, is given space to breathe and bolstered with new evidence from adult education research. Rather than loosely advocate a ‘less is more’ approach to giving information, readers are reminded why relating new information to a person's understanding and beliefs is the most promising way to facilitate learning. The adult education research selected here suggests that people are unlikely to recall more than three to five pieces of new information in one session, however motivated and capable they are. Provision of new genetic information is related in terms of the degree of psychological threat. There is a solid and important message here: to inundate patients with relevant information does not make for competent genetic counselling.

Regarding a psychological and social focus in genetic counselling, the authors disabuse any “vague notion of offering support.” The various counselling approaches tell of concrete techniques that Genetic Counsellors use to convey risk, grasp opportunities for intervention and ultimately help patients make the best decision they can at a particular time point. For our patients, the quality of uncertainty may change, with new technologies shortening the time to a diagnosis, but overall quantity may not, as families move on to the next phase of living with a hereditary condition. This creates new issues, with a role for ongoing care. The book ends with an entreaty to Genetic Counsellors “to help guide patients and their families on their lifelong journeys.”

It is a US-centric narrative, which the authors acknowledge. From a UK perspective, this shows in some terminology, the historical lens and the descriptions around provision of healthcare. In other regions of the world, particularly with emerging genetic counselling practices, it is unclear how applicable the assertion is that “any attempts to measure the success, conduct, and outcomes of genetic counseling must refer back to [the United States National Society of Genetic Counselors] definition.”

Taken as a whole, this is a passionate account of the state of play with a broad view on the many changes shaped, weathered and predicted. *Advanced Genetic Counseling* does not assume to answer all of the questions on current practice, but sets them within a history and vision that provoke fresh thinking on recurrent themes: Do original definitions of genetic counselling stand up? How best to parse uncertainty with our patients? What is the role of genetic counsellors in a technologically new world order? This is a book that deserves to be read and referred to as the conversation and the practice evolve.

